# Operationalizing and automating Data Governance

**DOI:** 10.1186/s40537-022-00673-5

**Published:** 2022-12-10

**Authors:** Sergi Nadal, Petar Jovanovic, Besim Bilalli, Oscar Romero

**Affiliations:** Database Technologies and Information Management Group, Universitat Politècnica de Catalunya - BarcelonaTech, Barcelona, Spain

**Keywords:** Data Governance, Data Integration, Big Data, Metadata

## Abstract

The ability to cross data from multiple sources represents a competitive advantage for organizations. Yet, the governance of the data lifecycle, from the data sources into valuable insights, is largely performed in an ad-hoc or manual manner. This is specifically concerning in scenarios where tens or hundreds of continuously evolving data sources produce semi-structured data. To overcome this challenge, we develop a framework for operationalizing and automating data governance. For the first, we propose a zoned data lake architecture and a set of data governance processes that allow the systematic ingestion, transformation and integration of data from heterogeneous sources, in order to make them readily available for business users. For the second, we propose a set of metadata artifacts that allow the automatic execution of data governance processes, addressing a wide range of data management challenges. We showcase the usefulness of the proposed approach using a real world use case, stemming from the collaborative project with the World Health Organization for the management and analysis of data about Neglected Tropical Diseases. Overall, this work contributes on facilitating organizations the adoption of data-driven strategies into a cohesive framework operationalizing and automating data governance.

## Introduction

Big Data is no more a synonym for large volumes. After its first years, the focus has steadily shifted towards data integration concerns, also known as the data variety challenge.^1^ Now, the difficulty is how to overcome the data heterogeneity, both syntactic and semantic, in order to integrate the right variables enabling insightful data analysis and covering the most relevant decisional aspects [1]. Indeed, private and public organizations aim at crossing the right data to make the right decision. Unfortunately, most of the times the required data is not available inside the organisation and, therefore, there is a need to contextualise the internal data with external data [2]. More precisely, data ecosystems are getting more and more complex nowadays, with the so-called data deluge. Relevantly, this problem may even arise inside the same organization. As a matter of fact, different departments and areas may produce heterogeneous data not easily crossable. For example, an e-commerce platform gathering information about the sentiment raised by their products in social networks and collecting price information about its competitors to forecast customer churn; a retail company acknowledging the music played at a time and at a store, the calendar of events/holidays, and users and clothing trajectories (i.e., how clothes and users *move* inside the shop, for example, from the shelves to the fitting room or directly to the counter) to contextualize the sales reports; a salesperson combining the internal data with neighborhood statistics such as average income, age and gender balance to decide the selling strategy in a certain area, etc.

The challenge in all these scenarios is to govern the data lifecycle. That is, to know what data are available (at the level of variables), to know the semantic relationships between variables from different sources, or to trace data processing so that one may explain why a certain result is given. More precisely, according to [3], *governance refers to what decisions must be made to ensure effective management (i.e., making and implementing decisions) and use of IT and who makes the decisions (locus of accountability for decision making)*. In the last years, there has been substantial efforts to define IT governance and IT management, and currently [3] is a seminal book on that matter. However, few works tackle data governance despite its current relevance. These works (e.g., [4]) claim there is a need to dig deeper and distinguish IT from data governance. Specifically, they introduce the concept of data asset and redefine governance and management for this specific asset.

**Fig. 1:**
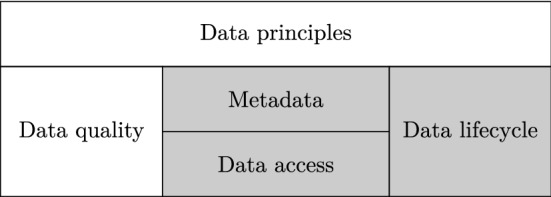
The organization data assets from [4] (in gray those covered by this paper)

Accordingly, data governance may be defined as to *what decisions must be made to ensure effective data management and data usage and who makes the decision (locus of accountability for data assets)*. Indeed, [4] argues that data governance is still a broad area covering data definition, management and accessibility. Figure 1, presents their approach to data governance. The *data principles* establish the link between the data assets with the business. As a first step, it is essential that the organization data assets are elicited and standardized at the business level (i.e., including domain experts). This is a typically long and complex process that requires the participation of domain experts and IT designers in brainstorming sessions that must provide, as result, an overall view, at the business level, of the organization data needs [5]. Next, the *metadata* describes the elicited data assets, which must be specified in a precise and concise manner and in a machine interpretable format. To that end, [4] describes three main metadata artifacts. The *physical metadata* must include information about the data physical storage, needed in order to access and manipulate these data. The *domain-independent metadata* includes relevant information for its processing and automatic management (such as ownership, file format specific processing information, etc.). The *domain-specific metadata* are asserted at different organization levels and capture the day-by-day terminology used in the organization (such as business concepts, organization’s key performance indicators—KPIs, etc.), and thus represents the bridge between the physical data representation and the business layer. Benefiting from the asserted metadata, which guarantees standardized and interpretable data assets, the *data access* aspect makes data available for different purposes (typically different business questions coming from different departments) and according to different Service Level of Agreement (SLAs). This aspect enables data consumption and the analysis within the organization. Last, but not least, Fig. 1 introduces two traversal aspects. The *data quality* aspect focuses on making data meet the user requirements and guarantee the needed quality for its exploitation, while the *data lifecycle* acknowledges that data moves through different stages and keeps track of such transformations.

Despite the efforts made by the community on defining the foundations for data governance, the adoption of these principles is scarce among organizations. Indeed, the *operationalization* of data governance (i.e., the definition of the data processes and flows that implement its principles) is not carried out systematically across organizations, and each organization defines its own data architecture and associated data flows that best suit their requirements. This is, however, a complex task which requires a high degree of specialization (e.g., data stewards) and a large amount of resources. It is of no surprise that only big tech players have successfully deployed in-house solutions for data governance. Successful examples of such solutions are *Amundsen*^2^ (by *Lyft*), *Databook*^3^ (by Uber), *DataHub*^4^ (by LinkedIn), or *Artifact*^5^ (by Shopify). Furthermore, cloud data providers provide layered architectures as a service, such as *Hortonworks Data Platform*^6^ or *Delta Lake*.^7^ However, still the task of designing the data flows that transform and move data from one layer to the next one is left to data stewards.

Departing from the above discussion, the objective of this paper is twofold. On the one hand, we define a data architecture and set of data governance processes that allow to systematically ingest, transform and integrate data from heterogeneous sources and serve them to business users. To that end, we introduce a novel Data Lake architecture which is stratified into different zones inspired by [6]. Precisely, the Data Lake is composed of three zones that have a specific purpose. The *Landing Zone*, stores raw data as they are generated in the sources. Next, the *Formatted Zone* homogenizes all ingested data into a common data model (in this paper, the relational data model) to simplify the process of integration. Finally, the *Exploitation Zone* serves integrated and transformed data for descriptive and predictive data analysis, this is data in the form of *tables*, *Dataframes*, and *tensors* [7]. Then, we propose a set of executable data governance processes responsible of moving data into the Data Lake and between zones. The *Data Collectors* extract data from external data sources and store it inside the Data Lake, while the *Data Persistance Loaders* and *Data Formatters* move data to the *Landing Zone* and to the *Formatted Zone*, respectively. Thus, we propose a framework to operationalize data management for data governance. Our framework is framed by [4] and covers the *data lifecycle*, *metadata* and *access* stages as introduced in Fig. 1. On the other hand, the second objective deals with the automation of such data governance processes under a set of generic assumptions that cover a wide range of use cases. Precisely, and following the terminology in [4], we present the *physical, domain-independent and domain-specific metadata* artifacts that allow to represent data sources, target tables and their mappings. Using such metadata, we are capable of automating the execution of *Data Collectors* for periodically loading data into the *Landing Zone*. Next, and assuming that the schema semi-structured datasets is available (e.g., JSONSchema for JSON data), the *Data Formatters* automatically parse into relations and populate the target tables using the specified mappings, which are of the form *one-to-many* and allow to model different loading strategies (e.g., insert or update). The execution of all such processes is orchestrated using a set of logs, which guarantee there is no redundant data in the different layers and the overall consistency of the served data. To the best of our knowledge, there is no available solution that combines both operationalization and automation of data governance.

*Contributions* The lack of a proper framework to operationalize data governance makes small and medium companies to struggle when adopting a data-driven strategy. As for now, only big companies can afford the investment needed to operationalize data management for data governance. Hence, we make a step forward towards facilitating the adoption of data-driven strategies into a cohesive framework that operationalizes and automates data governance. Shortly, our contributions are as follows:

- We present a layered Data Lake architecture and a set of standardized data flows that aid on the systematic operationalization of data governance.
- We propose a concise set of metadata artifacts that allow to automate the execution of the abovementioned data governance processes under a set of assumptions.

*Outline* We discuss related work in “Related work” section. In  “Use case: The Fight Against NTDs at WHO (WISCENTD)” section, we present a real use case exemplifying the challenges to tackle and that will serve as running example throughout the paper. Then, “Operationalizing Data Governance” and “Automating Data Governance” sections represent the core technical contributions, where we respectively present our approach to operationalize and automate data governance. Finally, “Conclusions and future work” section wraps up the main conclusions derived from this work and outlines future directions.

## Related work

Incorporating the use of technology in the organizations has been (and is) a well researched topic. Indeed, [3] is a seminal work in this area that defines a comprehensive framework with key organizational assets to be governed in order to successfully attain corporate governance: human, financial, physical, intellectual property, relationship and information and IT assets. However, as noted in [4], the work of [3] does not distinguish between IT assets (which refer to technologies such as computers, communication and databases) and data assets. It is from 2010 onward that the first frameworks aimed at governing data assets emerged. These, refine previous works at the corporate and IT governance levels and fine-grain the aspects related to data. Indeed, they follow a natural transition: once corporate and IT governance has been properly defined with frameworks identifying and separating concerns (i.e., decision domains) there is a need to drill-down and further investigate specific aspects. Although data governance has not drawn as much research as corporate and IT governance yet, some efforts have already been made (e.g., [8–10]). In our work, we focus on the data governance framework introduced by [4], which we earlier discussed in Fig. 1. As main contribution, they adopt the framework presented in [3] and extend it to define the five decision domains related to data assets. This way, they provide a data governance definition and its link to IT governance. However, the resulting data governance framework, as also happens to the other related works mentioned above, is still defined at a very high level of abstraction and cannot be directly instantiated by data practitioners within the organization systems.

In the rest of this section, we summarize related work dealing with data governance in a similar manner to our objectives. In the current literature, we find three big families of approaches about data governance: (i) as means to resolve poor data quality, (ii) to ensure availability of specific data sets to remain compliant with regulatory or legal provisions, effective reporting and integrated customer management and (iii) as means to increase the value of data as organization assets and framed by the concepts of corporate and IT governance [4]. Our work belongs to the third family and covers data ingestion, processing and availability but aligned with the organization goals, which is a key difference between (ii) and (iii). Regarding (i), even if we acknowledge the relevance of data quality, this is a complex problem that we leave out of scope and plan to tackle in the future.

### Operationalizing data governance

When operationalizing data governance, the border with the concept of data management gets diluted. Data management is defined as *the features a database management system (DBMS) must provide: namely ingestion, storage, modeling, processing, querying, concurrency and recovery strategies* [11]. Although data management was traditionally bounded within the DBMS, with the arrival of Big Data and the concept of Data Lake [12], it is nowadays acknowledged it can span different software packages. Indeed, this generalization of the concept of data management is one of the reasons why data governance is a hot research topic nowadays. Data architectures aim at separating concerns and identifying relevant tasks to be implemented in a successful data governance protocol. Thus, we focus on data architectures as the means to operationalize data governance. The first attempts (e.g., the $\lambda$-architecture [13]) were high-level descriptions that introduced relevant concepts such as the ingestion, batch, real-time and serving layers, which were adopted and polished by subsequent proposals. Recent works dig deeper and acknowledge the need to the effective and efficient production of more complex systems. Here, we focus on two main lines of work: *pond architectures* [14], and *zoned architectures* [6, 15, 16].

*Pond data architectures* Here, datasets are stored in different data ponds based on their properties (e.g., structured, semi-structured, and unstructured data) or the way data are generated (e.g., from IoT systems with high velocity or running software applications). This approach stores datasets only once organizing them with regard to the type of data (e.g., textual data [17]) and regardless of its final purpose or user need, hence not permitting dynamic repurposing of the data.

*Zoned architectures* In such approach, all data will be stored in different zones depending on the degree of transformation or refinement, fitted for a specific purpose. In contrast to the previous kind of architectures, here multiple copies of data can be stored in different zones, enabling the repurposing of data for different data processing and analytics pipelines required by end users. There are many variants of the Data Lake zones typically including at least the three main zones, namely, a zone for dumping raw datasets, known as *Landing Zone*, *Transient Loading zone*, or *Raw vault*; a zone with standardized and refined data, known as Harmonized or Refined zone, and a zone where data are ready for their final use, known as Distilled or Consumption zone. In [15], the authors evaluate the adequacy of using Data Vault modeling techniques [18] for modeling a zoned data lake, structuring it into *Raw* and *Business Vaults* as a generic separation and then Use-case specific *Data Marts* resembling the Consumption zone. [6] evaluates the use of data lakes in practice and detects the need for zoning data lake into several zones, where besides raw data, more structured copies of data are also stored allowing faster access to data by the end users. They also discover Hadoop as the preferred platform for setting up a data lake, enabling the storage for wide variety of data formats and different usage scenarios. Some approaches also include multiple intermediate zones [16], like Trusted zone with standardized and cleansed data or Discovery sandboxes where data are available for data wrangling or discovery actions.

### Automating data governance

Once a data architecture is in place, the challenge is how to orchestrate the systematic execution of processes that ultimately yield the required data to end-users. To that end, recent works acknowledge the relevance of metadata to govern the data assets [19–21]. This has motivated a wealth of *semantic-aware* architectures, that leverage on metadata to (partially) automate data exploitation, and to aid users in their decision making processes. In parallel, there exist systems targeted to automate a specific task within the lifecycle (e.g., schema discovery, data cleaning, or data fusion), which we denote as *task-specific automated governance*. Additionally, there exist approaches for *domain-specific data governance*, which provide solutions for specific domains (e.g., politics [22], cyber-physical systems [23], etc.). Besides being specific for a domain and not easily adaptable to others, they still present data governance frameworks meant to be largely manual. Therefore, even if they operationalize data governance in these domains, they do not automate it. Hence, in this subsection, we review related works on the first two areas.

*Semantic-aware data architectures* Constance [21] is a system that automatically extracts structural and semantical metadata from the contents of a Data Lake. Such metadata are used to define a unified query interface over the data sources. An alternative is Goods [24], Google’s solution to manage their Data Lake, which crawls, indexes and integrates heterogeneous datasets. One of its distinguishing features is the relationship graph, which encodes automatically extracted relationships between datasets like containment, provenance or content similarity. Then, a search engine uses this metadata to enable the exploration of the datasets. Finally, [25] presents a metadata framework for *Data Lagoons*, which are a certain kind of Data Lake for IoT scenarios. The authors propose a broker-based architecture that leverages a metadata repository which models aspects such as infrastructure resources, datasets, security or cost models. The usage of this metadata allows to define logical operators on the underlying datasets, such as aggregations, cleaning or profiling.

*Task-specific automated governance* The Data Tamer system [26], focuses on the process of data curation. It adopts a wrapper-based architecture to extract data from the sources into *sites*, representing collections of key-value pairs. Additionally, by making heavy usage of machine learning techniques, it provides modules for schema integration, entity consolidation and record linkage. An evolution of Data Tamer is the Data Civilizer system [27]. Besides including the aforementioned functionalities it also maintains the linkage graph where all relationships between tables or keys are represented. This graph is used to compute queries that discover join paths with data cleaning operators. An alternative is the VADA [28] architecture, aimed to support the process of data wrangling (i.e., extracting, cleaning and collating datasets). VADA is a knowledge-representation system that, relying on the expressivity of the Datalog± description logic, provides services for schema matching, schema alignment, data fusion or data quality, among others.

### Discussion

As a summary of this related work study, we conclude that current state-of-the-art is either too high-level (i.e., reference architectures serve as blueprint on what tools should be considered depending on the organization’s requirements and do not describe the data flows among them), or very specific either to a task or a domain. For example, data discovery is a well-researched problem focusing on identifying interesting or relevant datasets that enable informed data analysis [29]. Similarly, data integration in the context of Big Data (e.g., [30, 31]) aims at largely automating this specific task. However, none of the current works tackling these problems automate data governance and only focus on that task, which they largely automate, but failing to effectively manage the organization data assets throughout its complete lifecycle. In parallel, several big tech companies have presented their in-house solutions to automate data governance, which we denote *task-specific automated governance*. *Amundsen* by *Lyft*, *Databook* by Uber, *DataHub* by LinkedIn, or *Artifact* by Shopify, among many others. However, most of these tools are either proprietary or simplistic approximations to the problem in the form of data dictionaries that do not cover the whole data lifecycle. On top of that, most of them are ad-hoc to the needs of the company creating it.

## Use case: The Fight Against NTDs at WHO (WISCENTD)

To exemplify our approach, we present a use case based on a real project, which will serve as running example, where data are used on the fight against Neglected Tropical Diseases (NTDs) at the World Health Organization (WHO). NTDs form a group of 21 diseases with different, sometimes very complex aspects, all having in common that they affect population typically from economically challenging, rural areas of the world. Depending on the disease (e.g., Chagas disase, Leishmaniasis), transmission routes can vary (e.g., congenital from a mother to a child, blood transfusion, organ transplantation, insect bites), as well as the causes of its spreading worldwide (e.g., high presence and reproduction of insects in endemic areas, traveling to endemic areas, migration flows, etc.). All this makes the control and eventual elimination and/or eradication of NTDs very challenging. This is a paradigmatic example requiring to cross internal and external data to meet the informational needs of an organization and for which our approach fits naturally.

Recently, WHO has initiated building a data infrastructure that enables the collection and comprehensive analysis of NTD-related data (i.e., WISCENTD^8^). In particular, two subsystems have been created, (1) *WHO Integrated Data Platform (WIDP)*,^9^ which is powered by District Health Information System 2 (DHIS2),^10^ used to provide support for routine surveillance of the case diagnosis, treatment and to standardize data collection of NTDs at countries as well as country’s reporting to WHO, and (2) *WHO Integrated Medical Supply System (WIMEDS)*,^11^ powered by BonitaSoft,^12^ used to facilitate the distribution of medicine and diagnostic kits for NTDs to countries, as well as to enrich data collection with epidemiological information related to cases detected and transmission routes. However, given that NTDs have been typically overlooked by the national health information systems in the past, currently, relevant historical data are either non-existent or they must be searched elsewhere (e.g., non-governmental organizations, researcher repositories, United Nations, etc.).

To create a more comprehensive epidemiological picture about the status of NTDs at a country or globally (e.g., discover epidemiological silence, studying comorbidity of diseases), WHO needs an effective infrastructure to capture both the data being collected through their in-house systems (i.e., WIDP and WIMEDS) and data from existing external data sources. For instance, they require part of United Nation’s data^13^ referring to countries’ population and immigration, which UN reports yearly. Notice that this is important for monitoring the main indicators about the disease, which are typically computed over the territory population or total number of potentially affected people. Lastly, in order to enable NTD data analysis for a specific geographical area (i.e., country with its lower administrative levels) or for a specific health facility, WHO requires to include additional master data in the form of geographical hierarchy. Such master data are stored separately within the WIDP system, where they are updated after being reported by a member country (e.g., changes in the administrative level organization, new health facilities, requiring data collection at the lower administrative level).

For the sake of simplicity, we focus on these three most relevant data sources for analyzing NTDs (WIDP with *diagnosis* and *treatment* data, and *geographical* master data, WIMEDS with *medicine request* and *shipment* data, and United Nation’s *population* and *migration* data). Hereinafter, we will use this use case to exemplify our technical contributions. In the following, we discuss the challenges raised during the development of WISCENTD, and the need to address them via operationalization and automation.

**Fig. 2:**
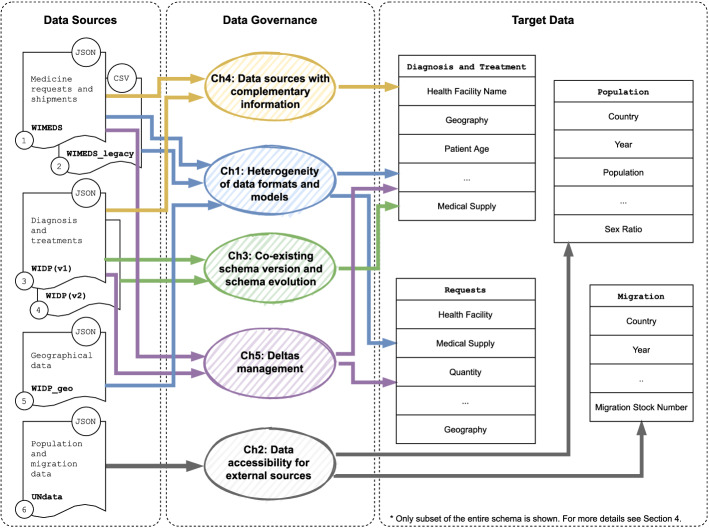
WISCENTD Use Case

### Data management challenges in WISCENTD

The data management challenges that arise in the WISCENTD use case, and which are addressed in our approach, are conveniently depicted in Fig. 2. Each challenge is represented with a shaded ellipse of a different color, where the incoming arrows indicate the data source(s) they arise from, and the outgoing arrows indicate what target (ready-to-be-consumed) data would their proper handling result in. Details and examples for each challenge follow next.

#### Heterogeneity of data formats and models

WISCENTD data sources consist of both structured and semi-structured data. Precisely, both WIDP and WIMEDS data are available in JSON formats (see for example an excerpt of diagnosis and treatment data of WIDP in Listing 1), while UN data are provided as CSV/Excel files.
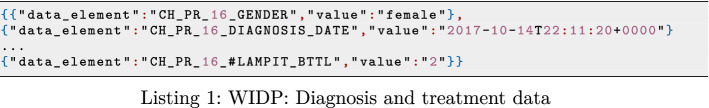


Semi-structured models like JSON provide high flexibility for structuring the data in different ways (i.e., replication and redundancy, nesting, cross-referencing, etc.), however they introduce certain complexity when it comes to data representation and its automatic processing. Take for instance the diagnosis and treatment data snippet, extracted from WIDP (see Listing 2) we can see that metadata used to create a data collection form (data_element) is mixed with data values. This is due to the data model provided by the underlying Web API of the DHIS2 platform, which is used to build WIDP. Unlike WIDP, in the case of WIMEDS, JSON format that stores the data coming from underlying *Bonitasoft* process management platform is free of other possible noise, allowing its automatic processing. See an excerpt of such data in Listing 2).
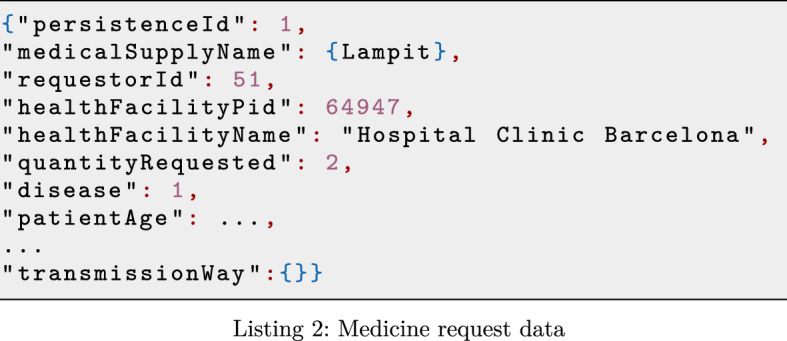


Handling the variability of different data models is a challenging task for data governance, while additional noise in form of application specific metadata could easily hinder the otherwise automatable data processing. Nevertheless, note that data sources typically expose the data in such a form as good habits promote and, in cases where they do not, their conversion to such form would require a simple source-specific pre-processing. For example, in Listing 3, we show a JSON snippet that resulted from converting the data in Listing 2, containing the same relevant information, but freed from additional unnecessary content (i.e., data_element and value keys).
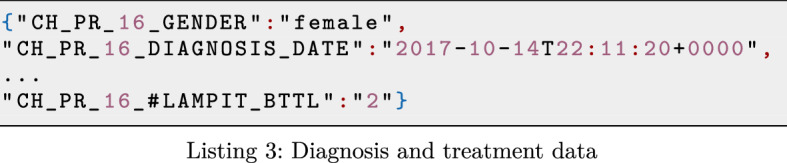


Moreover, having the additional metadata in form of a schema (e.g., JSONSchema) would allow automatic parsing of the a specific source format, thus making its data easily available for the rest of the data pipeline.

#### Data accessibility for external data sources

While the internal data sources can be ingested by dumping them periodically to the data lake, accessing external data sources can imply more complex processes, typically retrieving data via a publicly available API. Both WIDP and WIMEDS are accessed via Web APIs. The former is accessed by supplying at least programID that refers to the Web form from which the data should be obtained and lastUpdatedDate to specify the data period to which the ingested data refer. However, some APIs require more complex querying. For instance, the calls to the WIMEDS API require first to specify the business data type of interest (e.g., requests, shipments, medicines), and then, depending on the complexity of the information requested, a default or pre-defined custom business data query needs to be specified inside the API call (e.g., requests by disease, shipments by country, medicines by IDs).

UN data source (external to the WISCENTD system) is accessible via a publicly available API,^14^ by providing the artifact specifying the data category (in our case population and migration data) and artifactID specifying the variables to be retrieved (e.g., population totals, population by age range, population by gender, international migrant stock in absolute number or as percentage of population). Additional parameters can be provided to filter the results by specific countries or specific years.

From these examples we can see that accessing external data sources may sometimes require complex querying schemes that vary depending on the complexity of the exposed API. While the processing of specific data models could be generalized (e.g., reading of keys and values is common to mostly any JSON document), access patterns to obtain the data from the source would require source-specific programs, that can be parametrized to obtain the required portion of the input data (e.g., from specific endpoints or for specific time periods).

#### Co-existing schema versions and schema evolution

Moreover, being based on web forms, the schema of WIDP data is easily extensible to include new fields to be collected at countries. For instance, during the pandemic of COVID-19, another important parameter for treating Chagas disease patients could be whether they were previously infected by COVID-19, so that the potentially toxic and strong medicines for treating Chagas disease are administrated with care. In such case, a new variable (CH_PR_16_COVID19) is added to include such information to the WIDP data (see snippet in red in Listing 4).
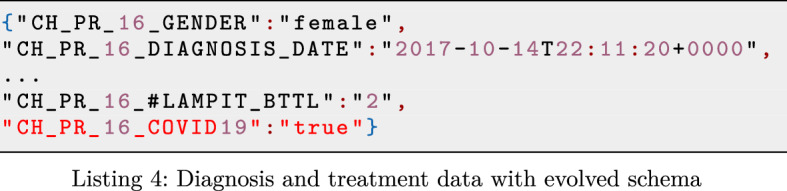


Although data formats like JSON allow to easily extend the schema to dynamically support modeling of the changing reality, handling the data with different schema versions is a challenging data governance task. To support the evolution of source schemata and allow the co-existence of different schema versions in the system, it is essential to maintain the metadata of different schemata and more importantly to guarantee the correct relationship between the data and its corresponding schema version.

#### Data sources with complementary information

Given that WIMEDS collects data about requesting medicines for treating Chagas patients (e.g., Lampit), it serves as a complementary data source to WIDP for treatment data. Such sources are important in the case of NTDs in order to create more complete picture about the current status of a disease in a territory and detect potential (intentional or unintentional) misreporting by the country.

This however introduces additional challenge in managing NTD data, given that it requires integration of such data and properly handling the possible duplicates. Importantly, proper data governance would require sufficient metadata to facilitate the integration of such data into a common dataset (e.g., conceptual model of the target domain and mappings of different source schemata to that model) and further enable other (instance-level) data integration steps like entity resolution.

#### Deltas during data ingestion

The lastUpdatedDate parameter in the WIDP Web API allows incremental ingestion of diagnosis and treatment data to the data lake, i.e., to ingest only the data reported after the previous run of the ingestion process. In WIMEDS, there is no system level parameter that enables incremental ingestion, however there are custom defined parameters that allow incremental ingestion per business data type (i.e., get requests by the date of request, get shipments by the date of shipment). Using the UN data API, we can at any time obtain a subset of data for specific time periods (i.e., years). However, geographical master data from WIDP, given their hierarchical structure, do not allow incremental ingestion (e.g., modification of intermediate administrative levels can obscure previously existing hierarchies and the update would require complex reconstructions). Thus, the ingestion of such data into a data lake is always complete.

Handling both incremental and complete data ingestion must be effectively supported in data governance systems in order to easily keep the needed freshness of the input data, while preventing unnecessary duplicates or inconsistencies in the data.

### On the need to operationalize and automate data governance

Following from the specific challenges detected in the WISCENTD use case, (but common to most Big Data projects), in this paper we propose a general framework to *operationalize* the data governance through a novel architectural proposal of a zoned Data Lake (inspired by [6]), as well as to *automate* complex data management tasks to drive the data through the complete data lifecycle, by means of concisely defined metadata artifacts.

Overall, the proposed framework allows an easy incorporation of internal and external data sources into data analysis pipelines, while effectively handling both structured and semi-structured data formats. New data sources are expected to be accompanied by minimum necessary metadata to automatically access, query, and parse their data. Such metadata can be either provided by the data source itself or automatically extracted using available *bootstrapping* techniques (e.g., [32]). The framework supports both incremental and historical (complete) data ingestion, accommodating thus a wide variety of source access schemes. Moreover, the framework provides effective mechanisms to deal with evolving data sources, enabling the propagation of source schema changes to the rest of data lifecycle. Lastly, by maintaining the correspondences between the data sources’ variables and a common (domain-specific) schema, the framework facilitates the integration among complementary or akin data, providing more complete and consistent view of the domain. In the following sections, we describe the proposed framework that operationalizes the data governance, as well as the automation of such framework under clearly defined assumptions, each denote as *Ax*.

## Operationalizing Data Governance

**Fig. 3:**
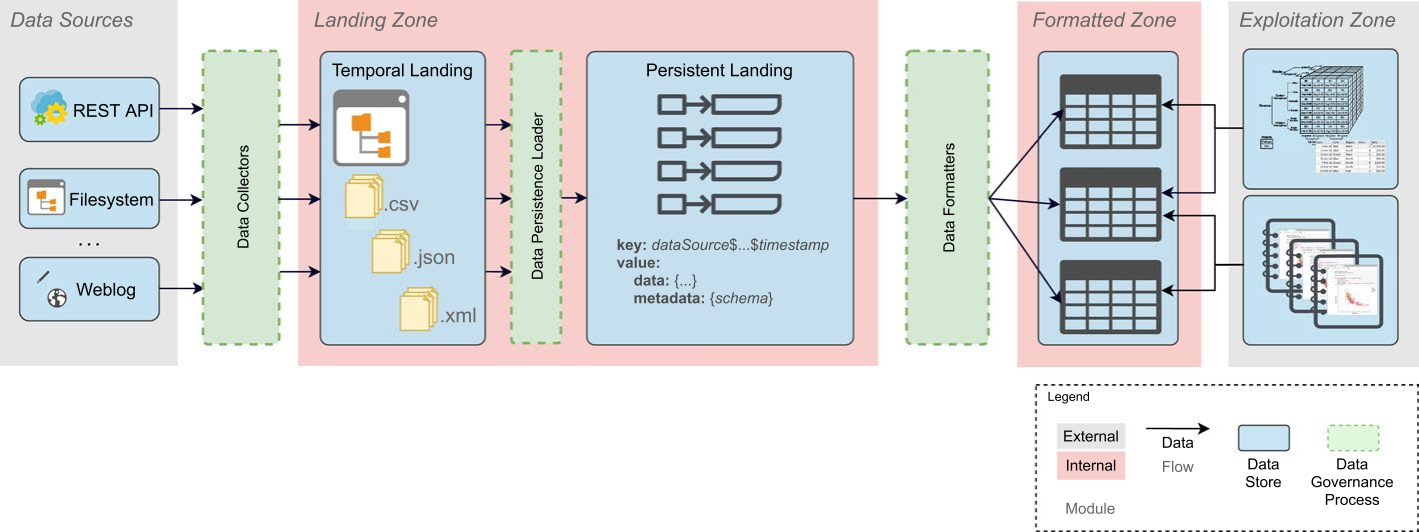
High-level overview of the proposed Data Lake architecture and its zones

In this section, we introduce the building blocks of our framework to operationalize data governance. The framework consists of a zoned Data Lake architecture that allows a clear separation of concerns and facilitates its instantiation towards different use cases. We additionally present the data flows, hereinafter, the *Data Governance Processes* (DGPs) that are responsible of moving data across the different zones. As shown in Fig. 3, the Data Lake is stratified into the (1) *Landing Zone*, (2) *Formatted Zone*, and (3) *Exploitation Zone*. Similarly, we distinguish between three kinds of DGPs, which manage the flow of data throughout the stratified Data Lake. We consider (1) *Data Collectors*, responsible of accessing specific data sources, extracting data from them, and moving the data into the data lake, (2) *Data Persistence Loader*, responsible of permanently and systematically storing the ingested data files, and (3) *Data Formatters*, which for each specific format of ingested data files, manage the conversion of the data into a unified format, making it suitable for the final consumption specific to the analytical needs of each user. In the following subsections we present and exemplify, for each zone, its functional description and discuss the DGPs responsible of the data flow.

### Landing Zone

The *Landing Zone* serves as entry point to the Data Lake, enabling fast ingestion of raw data in their original formats. Two submodules compose the *Landing Zone*, the *Temporal Landing* and the *Persistent Landing*, which as their names denote respectively represent a temporal storage of the ingested data and a permanent one.

*Temporal Landing* This submodule temporarily maintains the files that are automatically collected from the data sources and ingested into the Data Lake. Precisely, the *Data Collector* DGPs fetch data from the sources (either in a push or pull fashion) and store them as files in a hierarchy of subdirectories, one per data source, in order to support their exploration. To facilitate fast ingestion of new data, the *Temporal Landing* submodule naturally builds on top of a (distributed) file system that allows rapid storage and easy search for data files.

#### Example 4.1

**Fig. 4:**
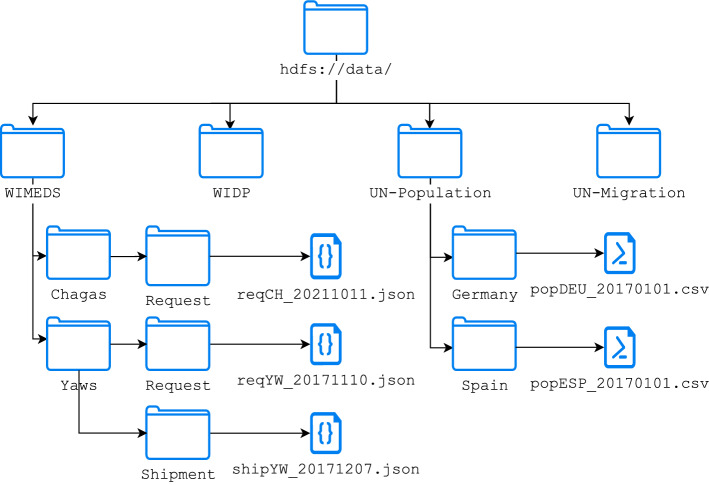
Example of the *Temporal Landing* for the NTD-related data

In our running example, the *Temporal Landing* component is deployed in a distributed file system (i.e., Apache HDFS), in order to provide both scalability for storing data files of possibly very large sizes and flexibility for storing data of different formats. To that end, data are conveniently organized in subdirectory structures specific to the domain (e.g., source being the root). In Fig. 4, we show an example of such hierarchies, focusing for simplicity only on the WIMEDS and UN-Population data sources.

*Persistent Landing* Once new data from the sources have been ingested and are available in the *Temporal Landing* submodule, the *Data Persistence Loader* DGP is in charge of systematically preparing them for facilitating the further integration process. To that end, such DGP must organize the newly ingested information and enrich it with additional metadata (e.g., schema definition), which must contain sufficient information to automatically parse the underlying datasets. To conveniently handle all this information, we assume *Persistent Landing is deployed using a Key-Value store (A1)*, on the one hand to permit easier access and fast querying of the stored data via *key*, and on the other hand, to provide flexibility in storing different information from the data source as *value*. More specifically, data still in their original formats, together with the collected or generated metadata necessary to automate further data processing, are stored as *value*, while the *key* is conveniently designed to enable efficient querying of such data and metadata. In particular, we compose the key such that the prefix determines the name of the data source, followed by the variable set of determinators specific for the source (e.g., format, data type, domain), and finally the timestamp as suffix. Such key design would enable fast querying of data from a specific data source, different data formats within the same data source, or ranges of consecutive extractions from the same data source.

**Fig. 5:**
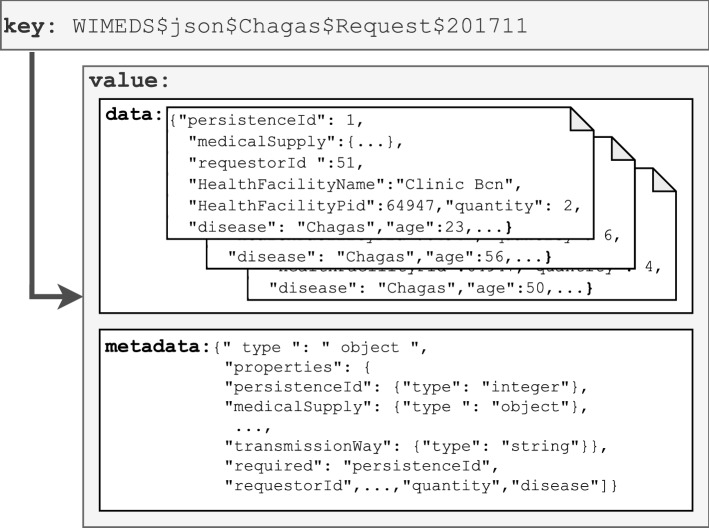
Persistent landing for medicine request data

#### Example 4.2

In our use case, we consider an Apache HBase data store as *Persistent Landing* submodule. There, WIMEDS data are stored using a generated composite *key* including the name of the source, source format (i.e., json), and a part of subdirectory hierarchy from *Temporal Landing* (e.g., *disease*, *data-type*, and *year-month*). Such key design allows for efficient range queries in HBase (i.e., access data in certain time periods and benefit from the distributed LSM-tree index cluster). For instance, to obtain all the medicine requests data for the Chagas disease, for the last quarter of year 2017, we run the following range query:
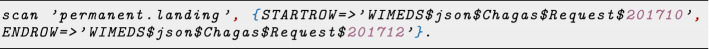


Furthermore, the HBase *value* includes two columns, *data*, with the content of the possibly multiple ingested JSON files of the data (e.g., medicine requests) from the WIMEDS data source, and *metadata*, the JSON schema referring to the ingested JSON files in the data column (see Fig. 5).

### Formatted Zone

The *Formatted Zone* contains a subset of ingested data, converted into a unified, structured format, enabling hence a homogeneous way to access and exploit the data from different data sources. To this end, the *Data Formatter* DGPs, one per data format (and independent of the data source from where the data originates) are executed to convert and transfer the data from the *Persistent Landing* submodule into the *Formatted Zone*. These DGPs use the accompanied schema information of the specific format (e.g., *.xsd* or *.jschema* files) to automatically process the data files and translate the data into a canonical data model. Precisely, we *assume a tabular format as canonical data model in the Formatted Zone (A2) *. Nevertheless, notice that this is not a strong assumption of our framework, but, it conditions the implementation of the *Data Formatter* DGPs.

Finally, once the data are converted and stored in the *Formatted Zone*, they are ready for further exploitation. Notice that these data, although reformatted, might still suffer from original data quality issues (e.g., missing values, inconsistencies, redundancy), which should be addressed depending on the final analytical purposes [33]. Addressing such data quality issues is out of the scope of this paper, but having the data uniformly structured largely facilitates data cleaning tasks [34].

#### Example 4.3

**Fig. 6:**
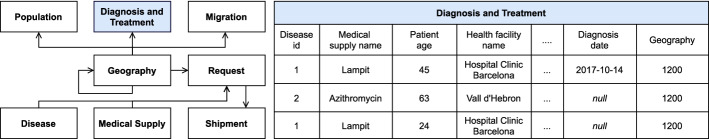
Example of the schema for the tables in *Formatted Zone* for the WIMEDS data

Retaking the running example, here, we deploy the *Formatted Zone* on a column-oriented DBMS (i.e., MonetDB), suitable for efficient OLAP-like operations. Thus, in the WISCENTD example, the medicine distribution data that were initially loaded as JSON files from the WIMEDS data source to the *Landing Zone* are now transformed into a set of relational tables inside the *Formatted Zone*. The resulting schema and a set of exemplary tuples corresponding to the *Diagnosis and Treatment* table are shown in Fig. 6. Note that the latter is fed with data coming from two different sources, that is from WIDP and WIMEDS (see “Data sources with complementary information” section).

### Exploitation Zone

Finally, the *Exploitation Zone* provides the analysis-ready data, to be consumed by end-users. Such data, depending on their exploitation purpose, are cleaned, integrated, (pre-)aggregated and exposed in the format suitable for further processing, analysis, and/or visualisation. We consider three exploitation models: *tables*, *Dataframes* and *tensors*, which are known to cover most analytical needs [7]. Therefore, this zone can host various views over the data previously stored in the *Formatted Zone*, each created potentially for a different data service. In this paper, however, we focus on the data management for governance and therefore we do not cover the details of producing different data views of the *Exploitation Zone*. Nevertheless, for the completeness of the proposed framework, we show how the systematic governance of data can facilitate final exploitation and analysis of data by different end users.

#### Example 4.4

The *Disease Programme Manager*, *Technical Officers* (epidemiologists, data managers), and *Mailing Office personnel* (administrative and logistic specialists) at WHO are interested in analyzing various aspects of history of medicine requests, shipments, or available manufacturer to entrust the medical supply procurement. To satisfy such requirements, data cubes are prepared to feed an OLAP tool that supports multidimensional analysis. In particular, the following three data cubes over the medicine distribution data are created to fulfill the requirements of such users:

- Cube 1: Describes a detailed view of medicine requests data, including important information such as *medicine requests process duration times*, the *requests status*, and the *geographic distribution of medicine requests*.
- Cube 2: Logistics information for mailing office personnel at WHO, such as the couriers doing the shipments and shipment process times. Such information could be also interesting for epidemiologists following the process at WHO and at the country.
- Cube 3: A cube that provides a link between specific medical supply and its available manufacturers, an information important for WHO Mailing Office personnel, who should choose the manufacturer to which the request order is entrusted.

**Fig. 7:**
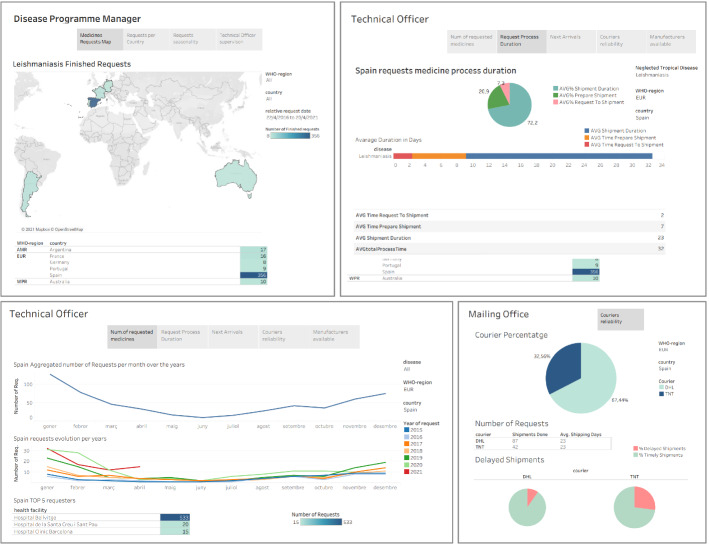
Example of the medicine request dashboards in the *Exploitation Zone*

These cubes are created over the *Formatted Zone* data previously stored in MonetDB. Given the requirement for multidimensional analysis of these data, for each cube, a star schema is created and implemented as a relational materialized view in an instance of PostgreSQL, which is specifically optimized for OLAP data access patterns, while MonetDB fully benefits of columnar data processing. Once created, such materialized views are then fed to *Tableau*, a visualization tool, and depending on the end user role (i.e., *WHO Technical Officer* or *Mailing Office Personnel*) several dashboards exist to analyze medicine distribution data (see Fig. 7). Similarly, a matrix containing historical data about the requests of a given country can be generated to allow for performing various advanced analysis. For instance, a time series analysis can be applied to check for trends, seasonality, or cyclic patterns, or even time series forecasting models can be applied to learn models that are able to flag countries based on their risk of *medicine shortage*. From a technological point of view, the creation of the exploitation zone boils down to conducting a traditional data warehousing project but with the huge benefit of starting from a single data source (i.e., the formatted zone), which largely facilitates its design.

## Automating Data Governance

In this section, we present our approach to automate data governance which boils down to the automatic generation and execution of DGPs. To that end, we rely on metadata artifacts modeling the components of our framework (e.g., data sources, how the data provided maps between zones, or the logs about the execution of DGPs). In the following subsections, we introduce in detail the considered metadata artifacts and the automated implementation of DGPs that exploit such artifacts considering the assumptions presented in “Operationalizing Data Governance” section.

### Metadata artifacts

Our proposal is based on [4], which, as depicted in Fig. 1, frames the data assets of a company. In our approach, *we assume the data principles have been previously elicited and elaborated (A3)* (e.g., by means of [5]). Thus, our contributions are: a systematic description of the *metadata* artifacts (*physical*, *domain-independent*, and *domain-specific*) required to execute the *data lifecycle* and *data access* aspects; and the implementation of generic DGPs that, using such metadata artifacts, automatically process and homogenize data incoming from evolving heterogeneous sources.

**Fig. 8:**
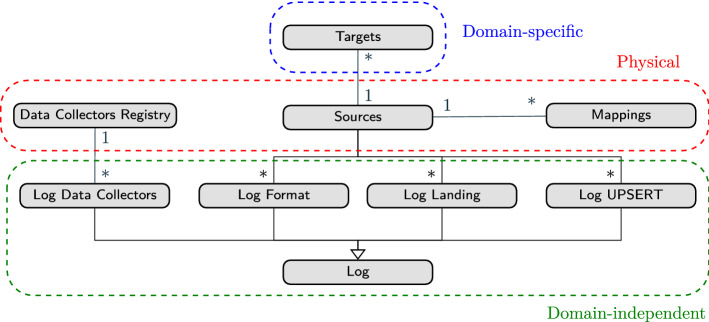
UML class diagram for the metadata artifacts

Figure 8, depicts a UML conceptual schema showing the metadata artifacts that enable automation of data governance in our framework and their relationships. Succinctly, we consider three kinds of artifacts implemented as tables. The *Physical* artifacts model how data are ingested into the Data Lake’s *Landing Zone* and the necessary information to automatically parse and integrate them into a unified view. Then, we consider a single *Domain-specific* artifact, which models the integrated information in the *Formatted Zone*. Finally, the *Domain-independent* artifacts, which are implemented as a set of logs, model aspects of the execution of the different DGPs..

**Fig. 9:**
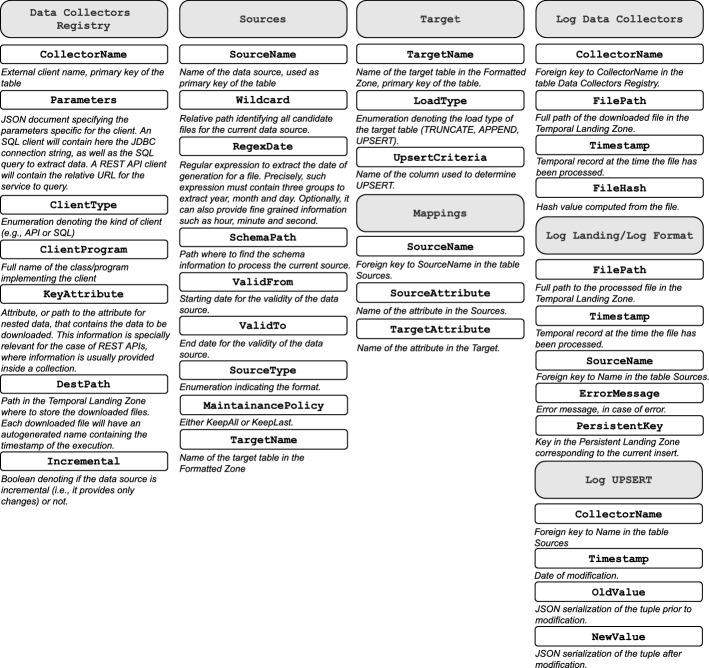
Metadata artifacts and their attributes

In our framework, *we assume that the Physical and Domain-specific metadata have been provided a priori (A4)* (i.e., either created manually or via the available automated approaches, while the *Domain-independent* metadata are automatically populated from the DGPs. Figure 9, concisely depicts and describes the attributes we define to govern the execution of DGPs. Throughout the rest of this section, we present a detailed description of them and the rationale behind their definition.

#### Data collectors registry

The DataCollectorsRegistry artifact maintains information about external data sources and where the data provided are to be stored. Examples of external data sources are REST APIs or relational databases. For each collector registered in this table, a new program will be automatically defined devoted to fetch data from an endpoint. To that end, *we assume the existence of custom code (i.e., the client) implementing the specific logic necessary to access each endpoint (e.g., authentication or protocol) given a set of parameters (A5)*. Examples of such parameters are the country code, for a collector fetching data from an API extracting migration data given a country. In this case, it would be required to define as many collectors as countries we want to extract data from, which boils down to add as many rows to the DataCollectorsRegistry table.

#### Sources

The Sources artifact maintains information about all data sources to be considered when loading data. This table, which is manually populated, allows to allocate new files stored in the *Temporal Landing Zone*, either loaded manually or via *Data collectors*. In this paper, we consider a data source to be that set of files that share the same schema version and data format for a continuous period of time. Thus, *we assume the existence of a schema file with the required information describing how to parse each source’s content (A6)* (e.g., an XSD file for XML data, or a JSON Schema for JSON data). For each source, we also encode its target table in the *Formatted Zone*, hence we do not consider that a data source maps to more than one target table. Note that the mappings between attributes are specified in the Mappings table, described below. An additional distinguishing feature of our approach is the inclusion of maintenance policies for the data to be stored in *Persistent Landing Storage*. We, precisely, maintain the following two types of security policies:

- *KeepAll*. All the historical data ingested in the Landing Zone is kept. This policy type is relevant for incremental data.
- *KeepLast*. Only the data corresponding to the last load is kept. This policy type is relevant for master data.

#### Target tables

The Targets artifact maintains the target table information in the *Formatted Zone*. We assume the *Formatted Zone* to adhere to the relational model, which must be manually defined a priori (e.g., to include integrity constraints). Some target tables will have a unique source, while others might have multiple ones. In order to have a general method that considers the specificities of each target table, we consider the following load types:

- *TRUNCATE*. Indicating that before loading the sources to the target table, its content must be removed.
- *APPEND*. Indicating that the loading of new data is carried out maintaining the history in the destination table.
- *UPSERT*. Indicating that a value must be updated if it already exists in the target table according to the UPSERT criteria attribute. If it does not exist, the value will be added as a new record.

#### Column Mappings

The Mappings artifact defines the mappings between attributes stored in the *Landing Zone* and their target attributes in the *Formatted Zone* tables. By default, we consider mappings between source and target attributes to be one-to-one matching the attribute names. For instance, let us assume a JSON data source with the keys *A*, *B* and *C*, and a target table with attributes *B*, *C*, *D*. Then, we would automatically map the common attributes *B* and *C*. In order to support the definition of more complex mappings (e.g., $A \mapsto D$), we consider the table Mappings allowing to define such kind of mappings.

#### Data collectors execution logs

Once a new file is generated and loaded from an external source, an entry is added in the LogDataCollectors metadata table. This stores basic information about the files that have been automatically loaded to the *Temporal Landing* submodule from data collectors declared in the DataCollectorsRegistry table.

#### Formatted and Landing DGPs logs

We define the artifacts LogLanding and LogFormat to store information about the process of extracting, integrating and loading data that the *Data Persistence Loaders* and the *Data Formatter* DGPs executions. Precisely, for each processed source file, a new temporal record is added indicating wether there has been an error or not. We additionally store the key that has been generated for that file in the *Persistent Landing Zone*.

#### Upsert logs

The artifact LogUPSERT maintains the necessary information to guarantee traceability of changes in the *Formatted Zone* when the UPSERT protocol is used. A new record is added to this table for each change applied by the UPSERT protocol, where the old and new tuple are serialized. In order to accomodate tuples conforming to multiple schemata we serialize them into JSON format.

### Automating DGP execution

**Fig. 10:**
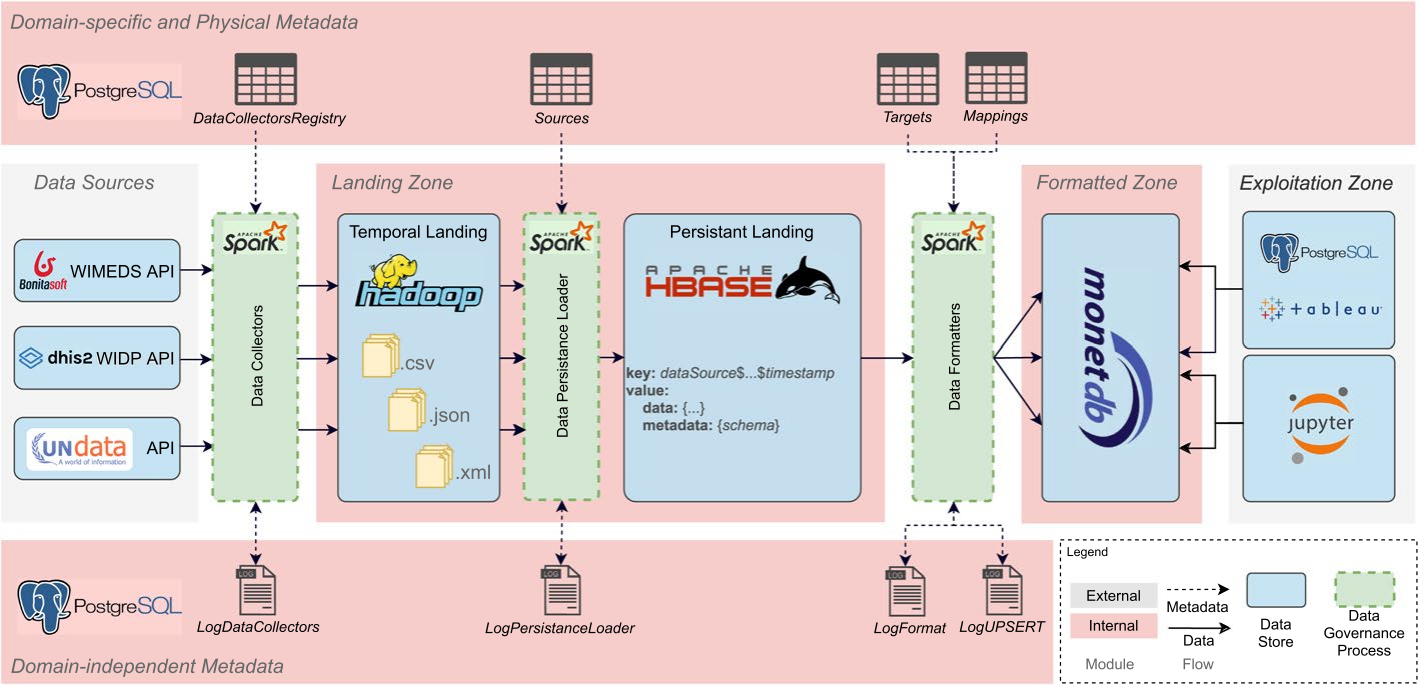
Instantiation of the system architecture for WISCENTD use case

Once the tables that will contain the infrastructure metadata have been described, we proceed to describe the DGPs (i.e., the data flows that will consume such metadata and automatically process data from the sources). First, in Fig. 10, we depict the instantiation of the proposed system architecture for the WISCENTD use case, used to showcase the particular components of our approach.

#### Data collectors

**Fig. 11:**
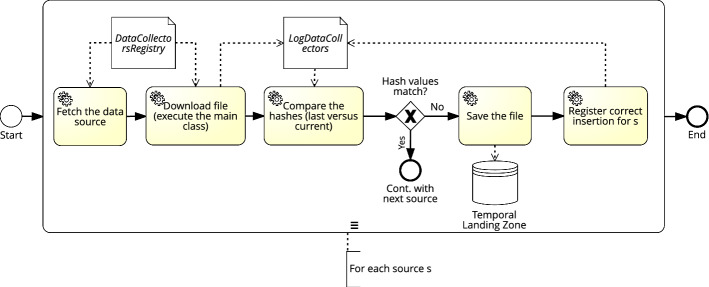
*Data Collectors* DGP

The process of downloading data from external data sources is governed by the DataCollectorsRegistry table (see “Data collectors registry” section). For each external source defined in the table, a *Data Collector* DGP is instantiated using the class indicated in the *ClientProgram* attribute. This class contains all the relevant code to access a specific data source and to extract the data indicated by the attribute *params*. Given that some sources are rather static (e.g., lookup information or master data), and in order to minimize the amount of duplicate data we store, we calculate the hash value of the downloaded file (e.g., using the *md5* algorithm). Such hash is compared to the latest download for that source, which is stored in the table LogDataCollectors (see “Data collectors execution logs” section). If both values match, then we ignore the current file as it is guaranteed to be exactly the same as the last download. Otherwise, a new log entry is added to LogDataCollectors. Figure 11, depicts the data flow of the *Data Collector* DGP that stores raw data in the *Temporal Landing Zone*.

##### Example 5.1

**Fig. 12:**
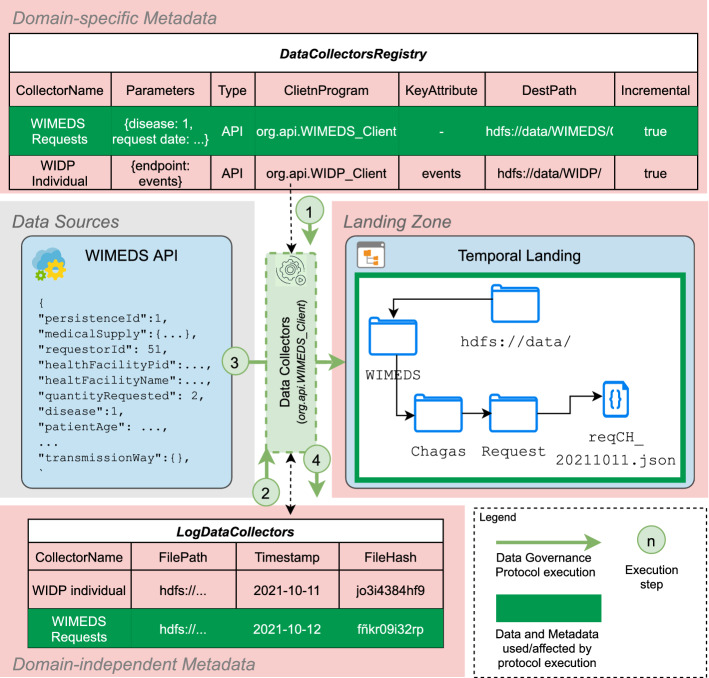
Execution of the *Data Collectors* DGP (WISCENTD Use Case)

The instantiation of the *Data Collectors* DGP for the WISCENTD use case is sketched in Fig. 12. The *Data Collectors* program processes the data sources registered in the DataCollectorsRegistry tables. In the case of WIMEDS, data are accessed via REST APIs and thus they are *pulled* using a set of prepared API queries. Yet, since many API endpoints, serving different types of data, are available, the program fetches the correct data using the *Parameters* provided in the DataCollectorsRegistry metadata artifact (step number 1). Once the data are extracted, the program compares the hash value of the newly fetched file with the corresponding hash value of the last fetch from WIMEDS (retrieved from the *LogDataCollectors* artifact, in step number 2). In our running example, the hash values are not the same, hence the new file is written to the *Temporal Landing* (step number 3), inside the corresponding directory. Otherwise, the process continues with its planned execution (i.e., fetch the following source, see Fig. 11). The directory hierarchy where the files are stored in the *Temporal Landing* is automatically created based on the information available in the *DestPath* attribute. Once the files are correctly written, a new row marking the successful fetch of the data is stored in the *LogDataCollectors* (step number 4), where among others the *path of the file* of the data stored in HDFS, and the *hash* value of the content are stored. Such information is necessary for the retrieval of the data from HDFS and for checking if the content is new (i.e., the hash value). Finally, notice that the data in the *LogDataCollectors* (including the *Timestamp*), can be used to trace back a data source stored in the *Temporal Landing*.

#### Data Persistence Loader

**Fig. 13:**
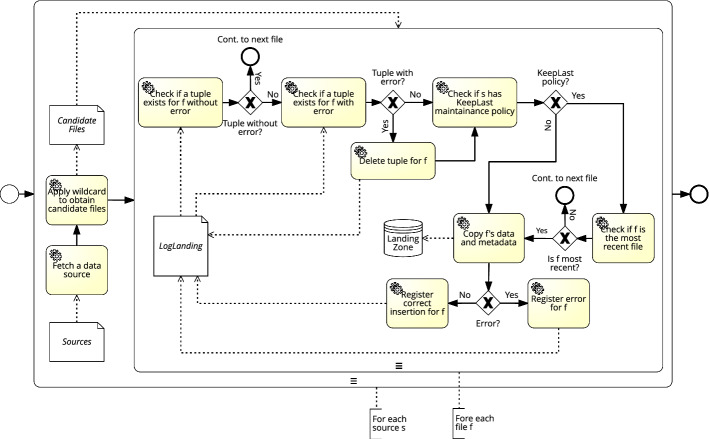
*Data Persistence Loaders* DGP

The loading of files from the *Temporal Landing Zone* to the *Persistent Landing Zone* is governed by the Sources metadata table (see “Sources”). For each source, a DGP will be generated applying its *Wildcard* and obtaining all files corresponding to that source. These are stored in the *Temporal Landing Zone*, and are candidates to be loaded to the *Persistent Landing Zone*. Querying the table LogLanding (see “Formatted and Landing DGPs logs” section) the DGP filters out candidate files that have already been processed. Those that have not been processed will be logged in LogLanding. Additionally, if the data source has a *MaintenancePolicy* of the kind KeepLast, a further filter will be applied to the candidate files so that only the most recent one is processed. Once the selected files have been processed, a new log entry will be added in LogLanding indicating the successful execution of the DGP, or the presence of an error. Figure 13, depicts the data flow of the *Landing Zone* DGP that processes and loads data from *Temporal Landing Zone* to the *Persistent Landing Zone*.

##### Example 5.2

**Fig. 14:**
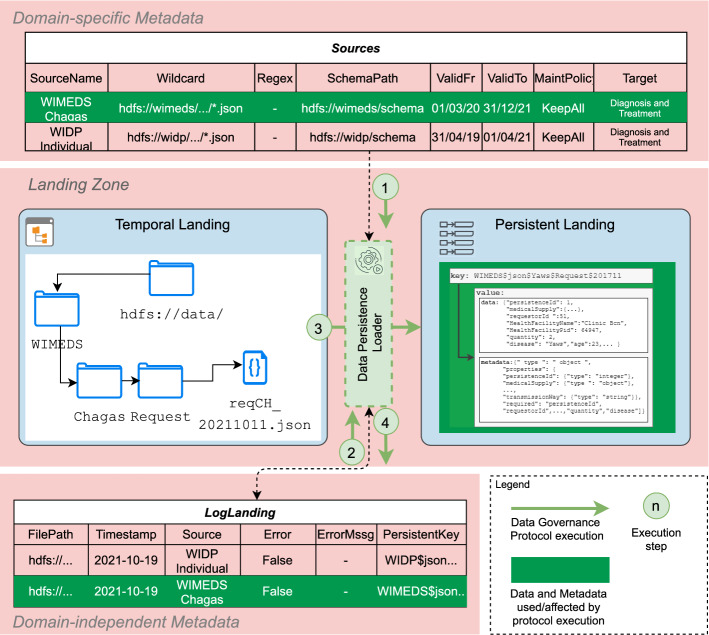
Execution of the *Data Persistence Loader* DGP (WISCENTD Use Case)

The instantiation of the *Data Persistence Loader* governance process for the WISCENTD use case is sketched in Fig. 14. In our use case, the WIMEDS directory in the *Temporal Landing* is organized in terms of Diseases and types of Requests (e.g., ad-hoc requests or forecasts for a longer period of time). The wildcard parameter in the Sources metadata artifact allows for the selection of all the files from the corresponding directory in HDFS (step number 1). For each file, a check of whether the file was previously loaded is done by consulting the LogLanding metadata artifact (step number 2). Since we assume this is the first execution and thus no data have been previously loaded, the data and the corresponding metadata are immediately loaded into an HBase instance in the *Persistence Landing* store (step number 3). To this end, the key of the HBase is defined as to optimize the retrieval of the data by internal processes (e.g., range queries), and uses the following structure Source, Disease, Request Type, Timestamp. Finally, once the data are stored as key values in HBase, the attempt of storing the data (i.e., successful or unsuccessful) is logged in LogLanding.

#### Data Formatters

**Fig. 15:**
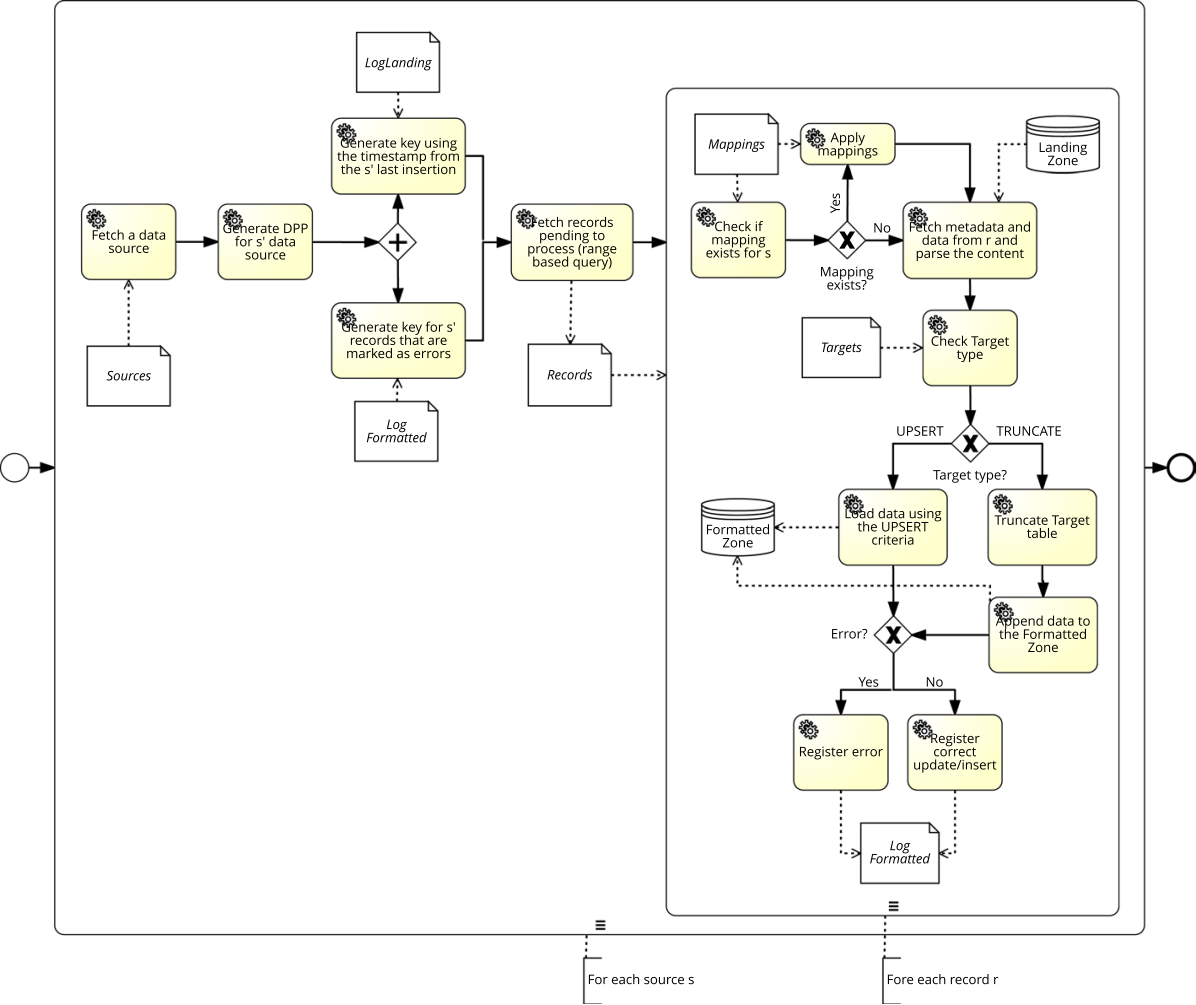
*Data Formatters DGP*

The final data processing step is the homogenization of data from the *Persistent Landing Zone* to the *Formatted Zone*, which is governed by the Targets metadata table (see “Target tables” section). To that end, for each target, the *Data Formatter* DGP generates a search key using the *timestamp* of the latest successful execution of the *Landing Zone DGP* for that source, which can be obtained from the LogLanding table (see “Formatted and Landing DGPs logs” section). Recall that the technological choice for the *Persistent Landing Zone* physically stores data in lexicographical order using the key, hence by defining a range starting at the timestamp of the last successful execution, we can fetch all files to be processed and integrated. Furthermore, by storing together their data and metadata (i.e., schema and parsing information), the population of the *Formatted Zone* can be automated regardless of the original format of data. During this phase, we also apply column mappings, which can either be based on matching attribute names, or using the Mappings table. Additionally, as previously mentioned, this DGP supports the *UPSERT* protocol. To that end, once the file to be loaded has been processed, using the *LoadType* attribute the DGP will decide the loading strategy, using the *Upsert Criteria* and logging each change to the LogUPSERT table (see “Upsert logs” section). Alternatively, if the target table is of kind *TRUNCATE*, then it will be first emptied followed by an incremental load. Figure 15, depicts the data flow of the *Data Formatter* DGP that integrates data from the *Persistent Landing Zone* to the *Formatted Zone*.

##### Example 5.3

**Fig. 16:**
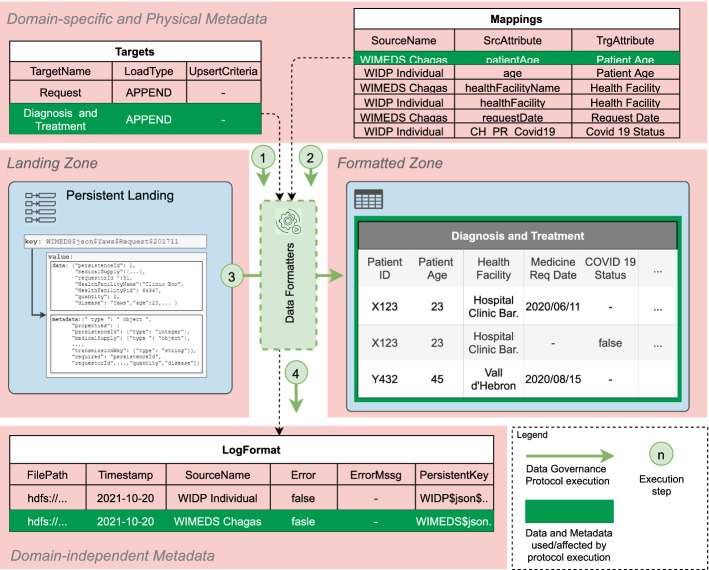
Execution of the *Data Formatters* DGP (WISCENTD Use Case)

The instantiation of the *Data Formatters* DGP for the WISCENTD use case is sketched in Fig. 16. In particular, in the *Formatted Zone* we initially design a Diagnosis and Treatment table that will be automatically populated via the *Data Formatters* process from two different sources, namely WIMEDS Requests and WIDP Individual data. Both of these sources contain information about patients and the treatments they receive. Therefore, in terms of diagnosis and treatment, these sources contain complementary information, and thus provide a more complete picture about the status of a disease in a given country. Furthermore, combining such information allows for detecting potential misreporting by the country (e.g., if medicine is requested via WIMEDS, but cases are not reported via WIDP). As can be seen in Fig. 16, in step number 1, the *Data Formatter* program uses the Targets metadata artifact to find the name of the table in the *Formatted Zone* and the type of loading to be performed (i.e., APPEND, TRUNCATE or UPSERT). Next, in step number 2, using the Mappings , the program knows that column *patientAge* from WIMEDS and column *age* from WIDP need to be mapped to column *Patient Age* in the target. Next, column *healthFacilityName* from WIMEDS and *healthFacility* from WIDP need to be mapped to *Health Facility*. Finally, the source specific columns *requestDate* from WIMEDS and *CH_PR_Covid19* from WIDP, need to be mapped to *Request Date* and *COVID 19 Status*, respectively. Since in this case the load type is APPEND, the program appends the corresponding data to the target table in step number 3, and because there are no errors during the execution, no error message is logged to the LogFormat, in step 4. Yet, other details like the *file*, *timestamp* and the *key* are logged in order to be able to trace back the source of the information.

## Conclusions and future work

In this paper we have followed two generic steps to address the two main identified challenges in data governance. First, operationalize the data management aspect (i.e., apply well-known data management practices), such as separating concerns, to systematically break down the data management problem into a set of effective and easily maintainable modules (i.e., the zoning approach). Second, we automate the processes that result from the operationalization (i.e., processes that deal with the flow of the data within zones) via DGPs.

### Threats to validity

In terms of threats to validity, in this paper we consider the following.

*Construct validity* Which refers to the extent that an experimental setting reflects the theory. Instead of experimental results, here we depict the proposed approach via a detailed example (i.e., the WISCENTD use case). The challenges defined in Sect. 3, arose during the development of the WISCENTD project, however they originally stem from the variety dimension of Big Data, and thus generalize to most Big Data projects. Hence, addressing them systematically, allows to adapt the framework in different use cases.

*External validity* Denoting the ability to generalize the obtained results beyond the presented setting. For this, it is important to consider we have made some assumptions that allow us to bound the scope of our method. Precisely, in Table 1, we show how each challenge was concretely addressed both in terms of operationalization and automation, and we additionally list the assumption(s) made at each stage. For the sake of an example, note that, in terms of operationalization, for *Ch1* — which had to do with the heterogeneity of data formats and models in the sources, the *Landing Zone* allows for an easy ingestion of any type of data, since it is based on a flexible data model (i.e., Key-Value store), and then the *Formatted Zone* allows for homogenizing the data for easy extraction. In terms of automation, the process of translating the data from one zone to the other is largely automated assuming that the schemas for parsing the contents of the data sources are available (*A5*).

**Table 1:** Challenges addressed in our framework, including what components provided *operationalization* and *automation* of data governance

Chall.$^*$	Operationalization	Automation	Assump.$^\dag$
*Ch1*	*Persistent Landing*, *Formatted Zone*	*Data Formatters DGP*	*A1,A2,A5*
*Ch2*	*Temporal Landing, Persistent Landing*	*Data Collectors DGP, Data Persitence Loader DGP*	*A5,A6*
*Ch3*	*Persistent Landing*	*Data Formatters DGP*	*A4,A6*
*Ch4*	*Landing Zone, Formatted Zone*	*Data Formatters DGP*	*A4*
*Ch5*	*Landing Zone, Formatted Zone*	*Data Formatters DGP*	*A5,A6*

$^*$ *Ch1* - Heterogeneity of data formats and models. *Ch2* - Data accessibility for external sources. *Ch3* - Co-existing schema version and schema evolution. *Ch4* - Data sources with complementary information. *Ch5* - Deltas management

$\dagger$ *A1* - Persistent Landing deployed as a Key-Value store. *A2* - Tabular format as canonical data model for the Formatted Zone. *A3* - Data principles previously elicited and elaborated. *A4* - Physical and Domain-specific metadata provided a priori. *A5* - Custom client code to access the source. *A6* - Schema file describing the source

### Future work

Through the WISCENTD use case we showed how data governance can be practically addressed via operationalization and automation. As future work, we aim to generalize our framework for supporting the specific needs of ML-based software systems (MLSS), namely those software systems which behaviour is greatly determined by ML models embedded therein. In this setting, the quality of incoming data, defined as *fitness for use*, is very low at its inception; therefore, a great deal of management, processing and analysis effort is required to increase its value. As a result, MLSS projects tend to be complex, inherently iterative and difficult to manage and govern, hence their systematic operationalization and automation is essential. Another promising research lines arising from our work is that of addressing the question on how to automate accountability from an automatically operationalized data governance, which requires collecting and analyzing provenance data about the process, data providers and data consumers.

## Footnotes

1 https://sloanreview.mit.edu/article/variety-not-volume-is-driving-big-data-initiatives/.

2 https://github.com/amundsen-io/amundsen.

3 https://eng.uber.com/databook/.

4 https://github.com/linkedin/datahub.

5 https://shopify.engineering/solving-data-discovery-challenges-shopify.

6 https://www.cloudera.com/products/hdp.html.

7 https://delta.io/.

8 https://www.who.int/neglected_diseases/disease_management/wiscentds.

9 http://quarry.essi.upc.edu:8083/wiscentd-wiki/index.php?title=WHO_Integrated_Data_Platform_(WIDP).

10 https://www.dhis2.org/.

11 http://quarry.essi.upc.edu:8083/wiscentd-wiki/index.php?title=WHO_Integrated_Medical_Supplies_System_(WIMEDS).

12 https://www.bonitasoft.com/.

13 http://data.un.org/.

14 http://data.un.org/ws/rest/{artifact}/{artifactId}/{parameters}.
